# The Optimized Fabrication of Nanobubbles as Ultrasound Contrast Agents for Tumor Imaging

**DOI:** 10.1038/srep13725

**Published:** 2015-09-03

**Authors:** Wen Bin Cai, Heng Li Yang, Jian Zhang, Ji Kai Yin, Yi Lin Yang, Li Jun Yuan, Li Zhang, Yun You Duan

**Affiliations:** 1 Department of Ultrasound Diagnostics, Tangdu Hospital, Fourth Military Medical University, 710032, Xi’an, Shaanxi, China; 2 State Key Laboratory of Cancer Biology, Department of Biochemistry and Molecular Biology, Fourth Military Medical University, 710032, Xi’an, Shaanxi, China; 3 Department of General Surgery, Tangdu Hospital, The Fourth Military Medical University, 710032, Xi’an, Shaanxi, China

## Abstract

Nanobubbles, which have the potential for ultrasonic targeted imaging and treatment in tumors, have been a research focus in recent years. With the current methods, however, the prepared uniformly sized nanobubbles either undergo post-formulation manipulation, such as centrifugation, after the mixture of microbubbles and nanobubbles, or require the addition of amphiphilic surfactants. These processes influence the nanobubble stability, possibly create material waste, and complicate the preparation process. In the present work, we directly prepared uniformly sized nanobubbles by modulating the thickness of a phospholipid film without the purification processes or the addition of amphiphilic surfactants. The fabricated nanobubbles from the optimal phospholipid film thickness exhibited optimal physical characteristics, such as uniform bubble size, good stability, and low toxicity. We also evaluated the enhanced imaging ability of the nanobubbles both *in vitro* and *in vivo*. The *in vivo* enhancement intensity in the tumor was stronger than that of SonoVue after injection (UCA; 2 min: 162.47 ± 8.94 dB vs. 132.11 ± 5.16 dB, P < 0.01; 5 min: 128.38.47 ± 5.06 dB vs. 68.24 ± 2.07 dB, P < 0.01). Thus, the optimal phospholipid film thickness can lead to nanobubbles that are effective for tumor imaging.

The emergence of molecular imaging is a milestone in the development of medical imaging. Molecular imaging allows the repeated, non-invasive and direct monitoring of the pathological processes of tumors; this monitoring can be performed dynamically and in real time at the cellular and molecular levels^1^. Ultrasound contrast agents (UCAs) for this branch of molecular imaging have been widely used in clinical trials, and they extend the diagnostic capability and utility of traditional imaging modes^2^,^3^,^4^,^5^,^6^,^7^. The current commercially available UCAs are usually designed to serve as blood pool agents with diameters of 1–8 μm^8^. Microsized UCAs that are used in molecular imaging have mainly been used in the intravascular imaging of inflammation, angiogenesis, plaque, thrombus, and similar states^3^,^6^,^9^,^10^,^11^,^12^,^13^,^14^. Microsized UCAs cannot pass through the vessel wall into the tumor tissue^15^,^16^,^17^,^18^,^19^,^20^,^21^,^22^. To overcome this limitation, nanosized UCAs have attracted considerable research attention due to their potential for extravascular molecular imaging^23^. Nanosized UCAs are applied to tumor-targeted imaging and therapy because the vascular endothelial gap in tumors is approximately 380–780 nm, which is much wider than that of normal tissue with a vascular endothelial gap of less than 7 nm.

Many studies have reported the preparation of nanosized UCAs^24^,^25^,^26^. Of these, UCAs composed of a phospholipid shell and a gas core, which are deemed nanobubbles and are fabricated using a thin-film hydration method, have shown optimal contrast enhancement abilities^27^,^28^,^29^,^30^,^31^. Compared with microsized UCAs, nanobubbles are better suited for targeted molecular imaging and may provide therapeutic benefits due to their small size^30^,^32^,^33^,^34^,^35^,^36^. Although many methods have been developed to fabricate pure nanobubbles, the majority of the methods are unable to directly produce uniformly sized nanobubbles, and the nanobubbles must thus be separated from a mixture of microbubbles. The post-formulation methods include gradient separation by gravitational forces, physical filtration or floatation, and they require the addition of amphiphilic surfactants during preparation^37^,^38^. These post-formulation methods may affect the nanobubble yield and stability, contaminate the sample, and generate material waste^39^.

In this study, we attempted to confirm the feasibility of using the thin-film hydration method of nanobubble preparation without post-formulation manipulation by controlling the thickness of phospholipid thin films. The physical characteristics of nanobubbles produced using the phospholipid film that had the optimal thickness were investigated. The zeta potential, morphology, *in vitro* and *in vivo* imaging enhancement ability, and the cellular location of the prepared nanobubbles were investigated and compared with those of SonoVue, a commercial microsized UCA.

## Results

### Preparation of nanobubbles

**Figure 1:**
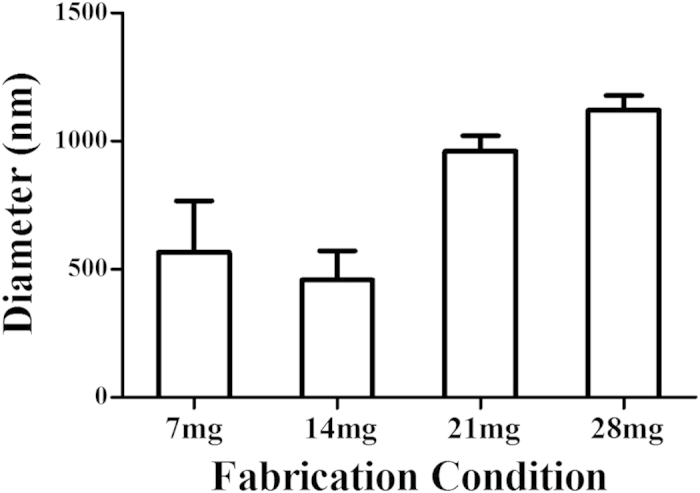
Histogram of the average diameter of the bubbles produced with 7 mg, 14 mg, 21 mg and 28 mg fixed-ratio mixtures of DPPC and DSPE.

**Figure 2:**
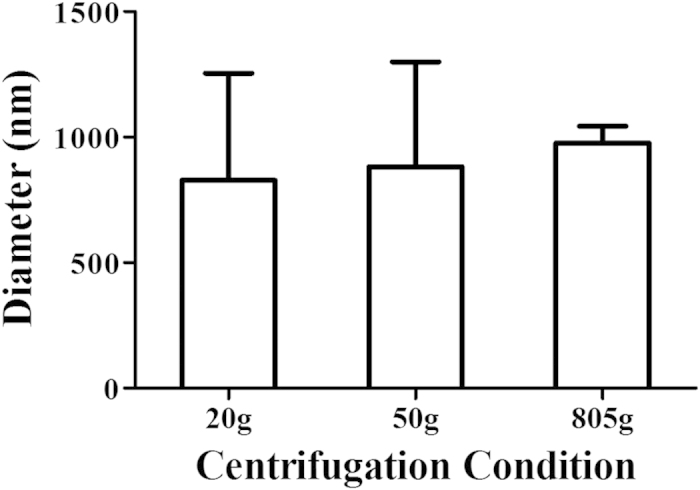
Histogram of the average diameter of the bubbles prepared using centrifugation speeds of 20 g, 50 g and 805 g.

The average particle size and zeta potential of the nanobubbles and SonoVue were measured by dynamic light scattering (DLS). The average diameter of SonoVue was 1614.8 ± 224.7 nm (n = 3), whereas the prepared bubbles had diameters of 565.2 ± 201.5 nm (n = 3), 457.9 ± 113.8 nm (n = 3), 960.8 ± 59.5 nm (n = 3) and 1121.1 ± 57.0 nm (n = 3), as shown in Fig. 1 and Supplementary Figure S1. The average diameters of the bubbles prepared using centrifugation speeds of 20 g, 50 g and 805 g were 828.4 ± 425.7 nm (n = 3), 882.1 ± 417.6 nm (n = 3) and 977.2 ± 65.9 nm (n = 3), respectively (Fig. 2 and Supplementary Figure S2). Zeta potential measurements showed that the nanobubbles produced using the optimal phospholipid film had a negative charge of −21.48 ± 7.46 mV (n = 3; Supplementary Figure S1 f) and those produced using SonoVue had a negative charge of −32.29 ± 13.13 mV (n = 3; Supplementary Figure S1 g). The statistical analysis indicated that there was no significant difference between the methods (P = 0.283).

### Nanobubble stability

The results of experiments on the nanobubbles prepared using the optimal phospholipid film suggested that the prepared nanobubbles had good stability. The average diameters of the nanobubbles stored separately at 25 °C for 1, 15, 30, 45 and 60 min were 457.9 ± 113.8 nm (n = 3), 504.3 ± 74.1 nm (n = 3), 519.5 ± 95.5 nm (n = 3), 625.9 ± 100.6 nm (n = 3) and 709.5 ± 272.0 nm (n = 3), respectively (Supplementary Figure S3 a–e). After 15 min of storage, the diameter distribution changed to bimodal; this change may have been caused by the rupture of some nanobubbles due to the formation of phospholipid complexes, resulting in the bimodal diameter distribution.

**Figure 3:**
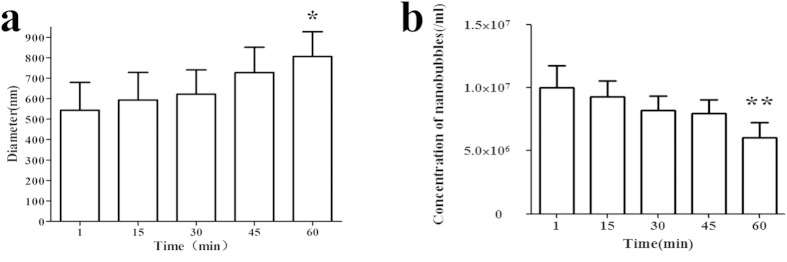
Histogram of the average nanobubble diameter (**a**) and concentration (**b**) changes over time (*P < 0.05, **P < 0.01 significantly different compared with values recorded at 1 min).

After 45 min of storage, the average diameter of nanobubbles was still approximately 600 nm (Fig. 3a), which was not significantly different from the average diameter after 1 min (P > 0.05). At 60 min, the nanobubbles became larger (P = 0.0121), but their average diameter remained under 1000 nm. The nanobubble concentration decreased over time; the concentrations at the 1, 15, 30, 45 and 60 min time points were 10.01 × 10^6^ ± 1.75 × 10^6^ per ml (n = 5), 9.31 × 10^6^ ± 1.22 × 10^6^ per ml (n = 5), 8.22 × 10^6^ ± 1.17 × 10^6^ per ml (n = 5), 7.96 × 10^6^ ± 1.09 × 10^6^ per ml (n = 5) and 6.07 × 10^6^ ± 1.20 × 10^6^ per ml (n = 5), respectively (Fig. 3b). However, the statistical analysis indicated that there was no significant difference between the 15, 30 and 45 min concentrations compared with that at 1 min; the only difference was for the concentration at 60 min.

### Nanobubble morphology

**Figure 4:**
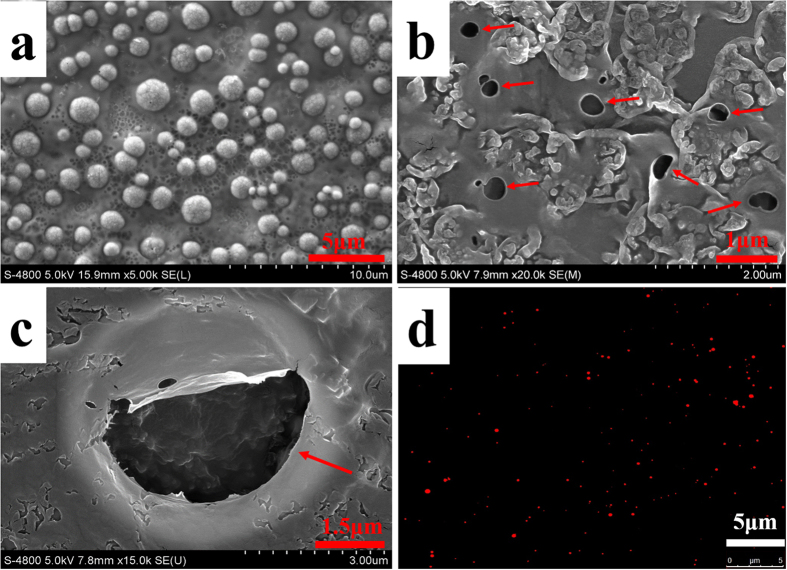
SEM micrographs showing the surface morphologies of the phospholipid (**a**), nanobubbles (**b**) and SonoVue (**c**). Fluorescence microscopy image of the nanobubbles (**d**).

In the scanning electron microscopy (SEM) micrographs, the phospholipid (negative control) appeared as solid spheres, whereas the SonoVue (positive control) and the nanobubbles were un-aggregated hollows (Fig. 4a–c). SEM indicated a size distribution of 200–650 nm for the nanobubbles, which is similar to the size distributions determined by DLS. The fluorescence microscopy observations also verified our results; nanobubbles with diameters <1 μm were directly observed (red dots, Fig. 4d).

### Nanobubble cytotoxicity assays

**Figure 5:**
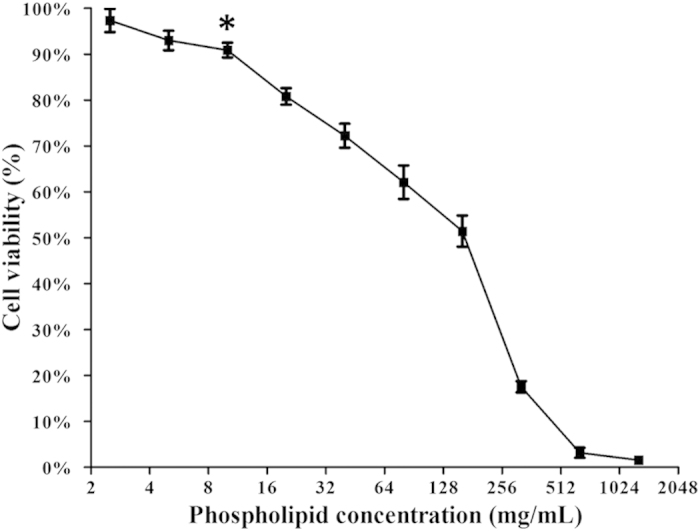
*In vitro* cytotoxicity for various phospholipid concentrations in the breast cancer cell line AU-565 determined using the MTT assay. Obvious cytotoxicity appeared when the concentration increased to 10 μg/ml (*P < 0.05 statistically significant difference compared with the initial concentration).

Because the nanobubbles are insoluble in cell culture medium, the nanobubble cytotoxicity was evaluated using the MTT assay. Figure 5 shows the cytotoxicity curve as a function of phospholipid concentration. Obvious cytotoxicity appeared when the concentration increased to 10 μg/ml. The results revealed that the nanobubbles had no obvious cytotoxicity to the AU-565 cell line for the phospholipid concentration (<5 μg/ml) that was used for the subsequent ultrasound imaging tests.

### *In vitro* ultrasound imaging enhancement ability

**Figure 6:**
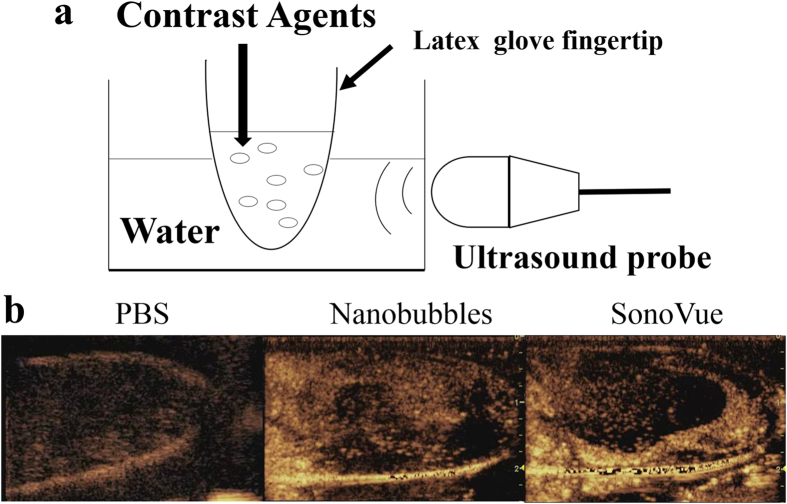
*In vitro* ultrasound image enhancement. (**a**) Schematic illustration of the *in vitro* experimental setup. (**b**) Ultrasound images of PBS, prepared nanobubbles and SonoVue using a 5 MHz probe.

The *in vitro* ultrasound enhancement ability of the prepared nanobubbles was compared with that of SonoVue; the specific detection method is illustrated in Fig. 6a. The nanobubbles showed good enhancement ability for ultrasound scanning (Fig. 6b). No significant difference was observed between the signal enhancements of nanobubbles and SonoVue (P = 0.691); the gray-scale intensity of the nanobubbles was 58.482 ± 28.192 dB (n = 5), while that of SonoVue was 52.861 ± 11.491 dB (n = 5).

### Nanobubble tumor imaging enhancement ability

**Figure 7:**
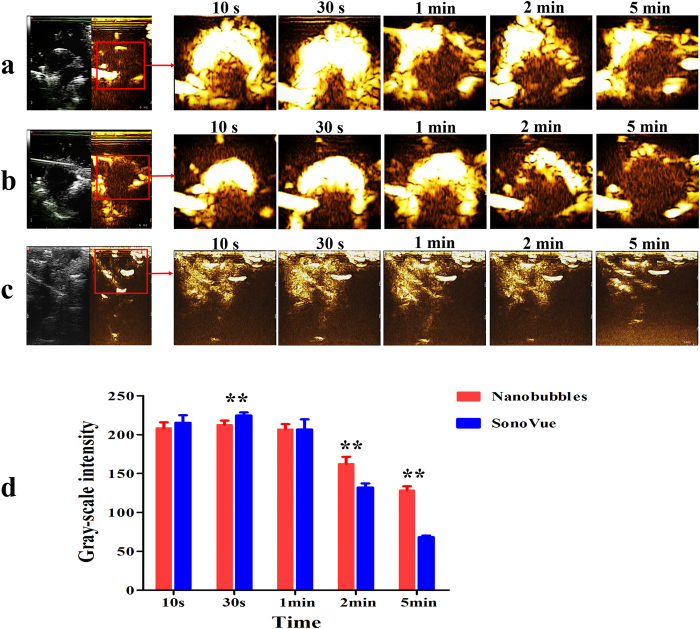
*In vivo* ultrasound image enhancement. Ultrasound-enhanced images of subcutaneous tumors before and after caudal vein injection of (**a**) nanobubbles and (**b**) SonoVue at 10 s, 30 s, 1 min, 2 min and 5 min. (**c**) Ultrasound-enhanced images of the liver before and after the injection of nanobubbles at the same time point. (**d**) Time-intensity histogram of the tumor gray-scale enhancement after caudal vein injection with nanobubbles (red) and SonoVue (blue). **P < 0.01 gray-scale intensity comparison between nanobubbles and SonoVue at the same time point.

To evaluate the tumor imaging enhancement ability of the nanobubbles, *in vivo* tumor imaging was performed on six breast tumor-carrying nude mice. No animals died during the experiments. Upon caudal vein injection, the enhancement of nanobubbles in the tumors increased over time. SonoVue was used as the control group, and images were recorded at 10 s, 30 s, 1 min, 2 min and 5 min (Fig. 7a,b). The imaging enhancement generated by the nanobubbles in the liver also shows an application of nanobubbles in organ imaging (Fig. 7c). The results suggest that nanobubbles are capable of serving as ultrasound contrast agents for tumors and organs.

Figure 7d shows a time-intensity histogram that compares the *in vivo* perfusion imaging features of nanobubbles and SonoVue. The gray-scale intensity increased immediately after a 150 μl injection of either nanobubbles or SonoVue. The time to peak intensity (TPI) for both the nanobubbles and SonoVue was 30 s. A statistically significant difference (P < 0.01) in the peak intensity (PI) of the gray-scale enhancement between nanobubbles (212.547 ± 5.414 dB; n = 9) and SonoVue (224.464 ± 3.969 dB; n = 9) was observed. After 2 min, the gray-scale intensity for the nanobubbles and SonoVue appeared to be significantly different (2 min: nanobubbles 162.47 ± 8.94 dB vs. SonoVue 132.11 ± 5.16 dB, P < 0.01; 5 min: nanobubbles 128.38.47 ± 5.06 dB vs. SonoVue 68.24 ± 2.07 dB, P < 0.01; n = 9). The imaging time is longer for the nanobubbles than for SonoVue.

### Location of nanobubbles

**Figure 8:**
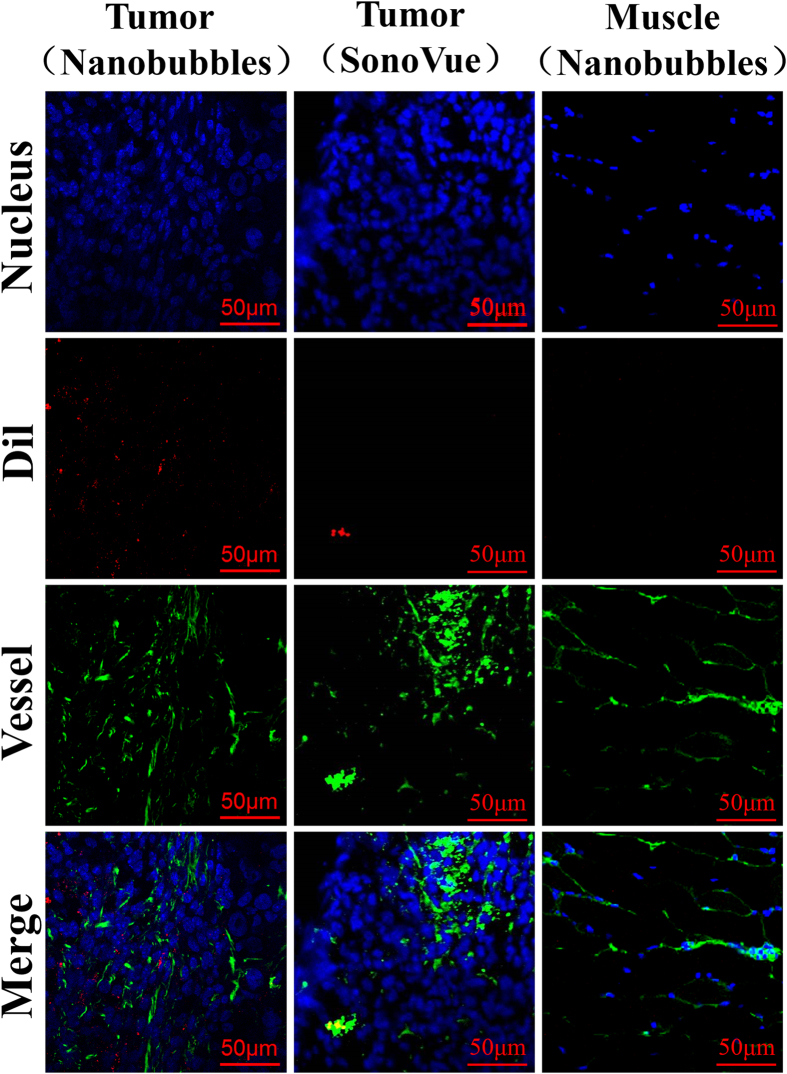
Confocal laser-scanning microscopy (CLSM) images of frozen pathological sections after nucleus and capillary labeling. Many DiI-labeled nanobubbles (red) were present in the extravascular and intercellular space of the tumor tissue (left); DiI-labeled SonoVue was hardly detected outside the tumor capillaries (middle), and DiI-labeled nanobubbles were not visible in the skeletal muscle sections (right).

The nanobubble locations after intravenous tail injections were confirmed using confocal laser scanning microscopy (CLSM) examination of frozen sections of tumor and skeletal muscle. The fluorescent dye for the vessels was green, whereas the nuclear dye was blue. In the present study, several DiI-labeled nanobubbles (red) were present in the extravascular and intercellular space of the tumor tissue, whereas DiI-labeled SonoVue was hardly detected outside the tumor blood vessels. In the skeletal muscle sections, however, DiI-labeled nanobubbles were rare (Fig. 8).

## Discussion

The particle size of UCAs is an increasingly important consideration in tumor imaging. Nanoscale bubbles are small enough to leak through the vascular endothelial gap to access the intercellular space in tumor tissue. We showed that the bubble diameters were determined by the preparation process, of which the most critical procedure was the thickness control of the 1,2-dipalmitoyl-sn-glycero-3-phosphocholine (DPPC) and 1,2-distearoyl-sn-glycero-3-phosphoethanolamine-N- [biotinyl(polyethylene glycol)-2000] (DSPE-PEG (2000)) phospholipid films. Minimally sized bubbles with good uniformity can be made using an optimal phospholipid film thickness; this film was formed by a 14-mg gross weight mixture of DPPC and DSPE-PEG (2000) dissolved in 2 ml of chloroform in a 25-ml rotary evaporation bottle. Because previous reports have indicated that nanobubble particle size also depends on the centrifugation speed during preparation^31^,^40^, we measured the average diameter and particle size distribution of the prepared nanobubbles, and we found that these values increased at centrifugation speeds of 20 g and 50 g. At 805 g, which means that centrifugation is not a key point during nanobubble preparation. The negative charge of anionic phospholipid DSPE-PEG (2000) nanobubbles can help prevent nanobubble aggregation, and the hydrophilic character can also keep the nanobubbles water dispersible^41^,^42^,^43^,^44^.

The fluorescence microscopy and SEM results provided direct observations of the nanobubble morphology and size. The nanobubbles observed with SEM did not appear to be spherical as previously reported^40^ because the bubbles ruptured in the vacuum environment during SEM scanning.

Obvious cytotoxicity appeared only when the concentration of DPPC and DSPE-PEG (2000) increased to 10 μg/ml. An MTT assay confirmed that the phospholipid concentration (<5 μg/ml) used to prepare the nanobubbles was nontoxic and was safe for further *in vivo* experiments.

The nanobubbles had a similar *in vitro* image enhancement ability as that of SonoVue. The echogenicity of nanobubbles was mainly due to its lipid shells, which allowed high scattering, as has been reported for other lipid-UCAs^45^,^46^. In *in vivo* experiments, the nanobubbles had a longer sustained tumor enhancement time compared with that of SonoVue. This result might be attributed to the small size of the nanobubbles, allowing the bubbles to enter the tumor tissue and not be washed out immediately. The DSPE-PEG (2000) lipid shells and the small size of the nanobubbles could prevent the nanobubbles from being cleared by the reticuloendothelial system^47^,^48^,^49^,^50^, which would help them pass through the relatively wider gap of the vascular endothelium and enter the intercellular space. The DiI-labeled nanobubbles were mainly found around the blood vessels, providing additional confirmation that nanobubbles leak through the pores of tumor vessels and then accumulate in the intercellular space through the vascular endothelial gaps in the tumor tissue. DiI-labeled nanobubbles were not detected in muscle tissue because the vascular endothelium gap of normal tissue is less than 7 nm.

In summary, the approach of modulating the thickness of the phospholipid film is feasible for the preparation of uniformly sized nanobubbles. The physical characteristics guaranteed a good imaging-enhanced ability of nanobubbles *in vitro* and *in vivo*. Currently, microsized UCAs play an important role in intra-vascular imaging, especially for highly vascularized organs or tumors. For poorly vascularized tumors, nanosized UCAs should have better performance in contrast-enhanced imaging. These advances in nanobubbles may be a step toward a new strategy in breast cancer patients from both the diagnosis and treatment points of view.

## Materials and Methods

### Materials

The DPPC (Mw = 734.039) and DSPE-PEG(2000) biotin (Mw = 3016.781) phospholipids were purchased in powder form (Avanti Polar Lipids Inc., Alabaster, AL) and used without further purification. Octafluoropropane (C_3_F_8_) gas was purchased from the R&D Center for Specialty Gases at the Research Institute of Physical and Chemical Engineering of Nuclear Industry (Beijing, China) and used as the filling gas for the nanobubbles. Phosphate-buffered saline (PBS) was purchased from GIBCO (Grand Island, NY). The fluorescent probes DiI and DAPI were purchased from Beyotime (Haimen, China).

### Fabrication of nanobubbles

Fixed-ratio mixtures of the phospholipids DSPE-PEG (2000) and DPPC (7, 14, 21 or 28 mg) were added to 25-ml rotary evaporation bottles and dissolved in 2 ml of chloroform. A small amount of the fluorescent membrane probe DiI (red fluorescence) was then added. Rotary evaporation was performed for 10 min at 55 °C and 120 rpm/min in a rotary evaporator (New Brunswick Scientific, Enfield, CT, USA). After the chloroform evaporation, milky white phospholipid thin films were observed on the rotary evaporation bottle walls. The milky white phospholipid thin films were hydrated with 0.5, 1, 1.5 or 2 ml of hydration liquid consisting of 10% glycerol and 90% 1 × PBS (V/V). The rotary evaporation bottles were placed in an incubator-shaker (New Brunswick Scientific) at 37 °C and 130 rpm for 60 min. Then, 500 μl of each suspension were transferred into four vials sealed with plastic caps. The air in the vials was replaced with C_3_F_8_ gas using a 50-ml syringe with a long, fine needle. Finally, every vial was oscillated for 45 s in a mechanical oscillator (Ag and Hg mixer, Xi’an, China) to generate the bubbles. The bubbles in each vial were separately diluted to 8 ml in PBS. All bottles and vials were covered with aluminum foil to prevent fluorescence quenching. In addition, commercial SonoVue microbubbles were used as the control.

### Particle size and zeta potential measurements

The particle sizes and zeta potentials of the nanobubbles and SonoVue were determined using DLS (Beckman Coulter, USA) with a laser wavelength of 660 nm at an angle of 90° using photon correlation spectroscopy (PCS) at 25 °C; 2 ml of diluted bubbles from each group and SonoVue were placed separately in sample wells. Based on the results of the particle size measurements, the ideal sample was identified as the one with the smallest average diameter that did not contain microbubbles; this sample, along with SonoVue, was used in the zeta potential measurements. The particle size and zeta potential of each sample were measured three times.

### Influence of centrifugation on the bubbles

The particle size measurement results indicated that the three non-ideal nanobubble samples (fabricated using the 7-, 21- and 28-mg fixed-ratio mixtures of DSPE-PEG (2000) and DPPC) contained microbubbles. Three centrifuge tubes were each filled with 3 ml of the nanobubbles fabricated using the 21-mg fixed-ratio mixture of DSPE-PEG (2000) and DPPC. Centrifugation was performed separately for each tube at 20, 50 and 805 g (relative centrifuged field, RCF) for 5 min. The nanobubble particle size in each tube was tested as described above and measured three times.

### Nanobubble stability

The change in the nanobubble particle size and concentration over time was measured using the ideal nanobubbles. All samples used for stability measurements were prepared under the same conditions as those used for particle sizing. The particle size of 1 ml of diluted nanobubbles was measured immediately after the completion of fabrication at 25 °C, and the remaining nanobubbles were kept at 25 °C. The particle size of the remaining nanobubbles was then measured after 15, 30, 45 and 60 min. At each time point, the nanobubbles were concurrently transferred to a hemocytometer for cell counting. Three images per sample (400x) were acquired at random locations using an inverted fluorescence microscopy (Carl Zeiss, Oberkirchen, Germany). These experiments were repeated three times, and the presented histogram of particle sizes and concentrations are based on the number-averaged calculations.

### Scanning electron microscopy

The first few steps used to fabricate phospholipid suspensions are same as for the nanobubble fabrication process; however, the air in the vials was evacuated using a 50-ml syringe with a long, fine needle, and C_3_F_8_ gas was not infused. The vials were then oscillated for 45 s. Without the infusion of C_3_F_8_ gas, the milky white suspensions did not form nanobubbles, and the phospholipids were retained. A single drop each of the phospholipid suspension, nanobubbles and SonoVue were separately loaded on dust-free foil and placed in a desiccator. After the solvent evaporated, each sample was gold sputter-coated for 5 min. Field emission scanning electron microscopy (HITACHI S-4800, Japan) was conducted with a gun acceleration voltage of 5.0 kV.

### Fluorescence microscopy

A suspension of DiI-labeled nanobubbles (200 μl) was diluted to 2 ml (1:10) with PBS, and a drop was placed between glass slides for fluorescence microscopy examination (100 × oil objective lens, Zeiss Axioskop, WEL Instrument CO. LLC). The fluorescence images of nanobubbles were then recorded with a digital camera (Zeiss Axiocam MRc5).

### Cytotoxicity assay

Cells from the AU-565 human breast cancer cell line were seeded in 96-well plates at a density of 6,000 cells/well with 100 μl/well of RPMI-1640 medium containing 10% fetal bovine serum (FBS). The cells were then cultured for 24 h in a humidified atmosphere with 5% CO_2_ at 37 °C. Subsequently, the cells were continuously cultured for 24 h in the same volume of fresh medium containing various concentrations of phospholipids (2.5–640 μg/ml). The medium was then replaced with 100 μl of fresh medium containing 10 μl of MTT solution (5 mg/ml), and the cells were incubated for 4 h. After the MTT-containing medium was discarded, 100 μl of dimethyl sulfoxide was added to each well to dissolve the substrate. After low-speed oscillation for 10 min, the absorbance of each well at 494 nm was measured using an Infinite F200 multimode plate reader (Tecan, Männedorf, Switzerland).

### *In vitro* ultrasonic-enhanced

A rubber glove finger containing 10 ml of 1 × PBS was placed in water and exposed to ultrasound (Fig. 6a). Ultrasound images were recorded on a MyLab Twice ultrasound system (Esaote, Italy) in visualization mode with a thyroid transducer at 5 MHz. After images were recorded of the PBS in the rubber glove finger, nanobubbles (200 μl) were injected into the rubber glove finger and images were recorded at 25 °C. Finally, SonoVue was injected into another rubber glove finger containing 10 ml of 1 × PBS, and ultrasound images were recorded.

### *In vivo* ultrasound-enhanced tumor imaging

The cancer xenograft model was established by the subcutaneous injection of 5 × 10^6^ MDA-MB-231 tumor cells into the right side dorsal scapular area of female athymic nude mice. Approximately 2 to 4 weeks after inoculation, based on the caliper measurements of perpendicular dimensions, the tumor volume was determined to be approximately 700 to 1000 mm^3^. All animal experiments were performed according to the guidelines issued by the local committee on Animal Care and Use, which are in accordance with the National Institutes of Health (NIH) guidelines. The ultrasound-enhanced tumor images were recorded using an Esaote MyLab Twice ultrasound unit with a broad bandwidth (3–9 MHz) routine clinical linear array transducer (Esaote, LA522). All images and films were recorded as digital files for subsequent playback and image analysis. Nanobubbles (150 μl) were injected into each mouse under isoflurane anesthesia through the tail vein, and the enhanced tumor and liver images were recorded immediately after injection until the end of the enhancement. After the nanobubble imaging, the nanobubbles were allowed to clear the circulation system of the mice for 2 h. Subsequently, the enhanced tumor images were recorded for SonoVue using the same volume and imaging protocol. Images were collected at 10 s, 30 s, 1 min, 2 min and 5 min post-injection for both nanobubbles and SonoVue. All digital clips and images were stored for gray-scale analysis, which was performed using WCIF ImageJ software (v1.37; National Institutes of Health, Bethesda, MA). Time-intensity curves for the nanobubble and SonoVue images were created and analyzed statistically using SPSS software (v19.0; SPSS Inc., an IBM Company).

### Pathological section

DiI-labeled SonoVue was prepared first; 2 μL of DiI (1.2 mg/ml) was added to 1 ml of SonoVue suspension. After 5 min, the mixture was centrifuged at 20 g for 3 min. We then collected the supernatant suspensions and verified the fluorescence of SonoVue MB under fluorescence microscopy. The nude mice bearing MDA-MB-231 breast cancer xenograft tumors were sacrificed after the injection of nanobubbles and SonoVue. Tumors were immediately separated to obtain frozen sections. The right thigh muscle tissue samples of the mice injected with nanobubbles were used as the negative control. Frozen sections were incubated with Isolectin-b4 (1:1000, Beyotime, Haimen, China) for 8 h at room temperature and washed three times with 1 × PBS. The sections were incubated for 2 h with anti-biotin as a secondary antibody (1:1000, Beyotime, Haimen, China) and washed three times with 1 × PBS. DAPI was used for nuclear staining. Finally, the tumor sections were examined under CLSM.

## Additional Information

**How to cite this article**: Cai, W.B. *et al.* The Optimized Fabrication of Nanobubbles as Ultrasound Contrast Agents for Tumor Imaging. *Sci. Rep.* **5**, 13725; doi: 10.1038/srep13725 (2015).

## Supplementary Material

Supplementary Information
