# Supplementary material for: The Optimized Fabrication of Nanobubbles as Ultrasound Contrast Agents for Tumor Imaging
Source: Sci Rep. 2015 Sep 3;5:13725. doi: 10.1038/srep13725 (PMC4558543; doi:10.1038/srep13725)
Supplement: Supplementary Information [file srep13725-s1.pdf]

The Optimized Fabrication of Nanobubbles as Ultrasound Contrast Agents for Tumor Imaging

WenBin Cai[1a], HengLi Yang[1a], Jian Zhang[2], JiKai Yin[3], YiLin Yang[1], LiJun Yuan[1], Li Zhang[1*], YunYou Duan[1#]

[1] Department of Ultrasound Diagnostics, Tangdu Hospital, Fourth Military Medical University, 710032, Xi'an, Shaanxi, China.

[2] State Key Laboratory of Cancer Biology, Department of Biochemistry and Molecular Biology, Fourth Military Medical University, 710032, Xi'an, Shaanxi, China.

[3] Department of General Surgery, Tangdu Hospital, The Fourth Military Medical University, 710032, Xi'an, Shaanxi, China.

[a] These authors contributed equally to this work.

[*] Correspondence to zhanglicwb@hotmail.com

[#] Correspondence to duanyy@fmmu.edu.cn

Supplementary Figures

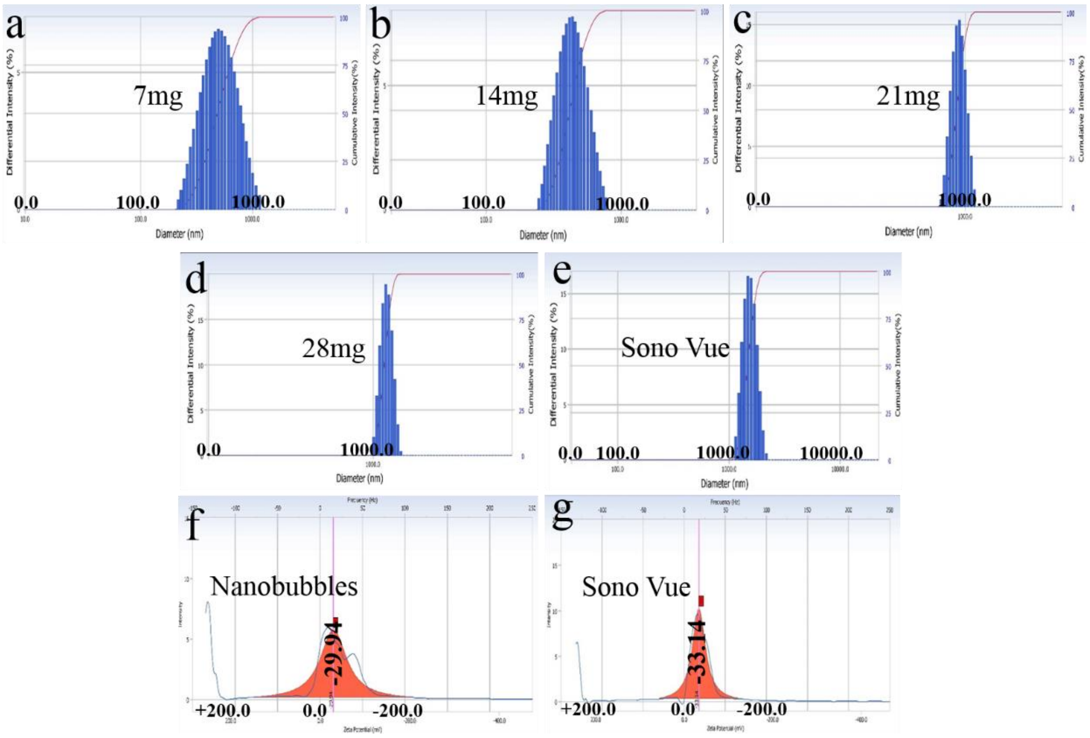

Supplementary Fig S1: Diameter distributions of the nanobubbles produced with 7 mg (a), 14 mg (b), 21 mg (c) and 28 mg (d) fixed-ratio mixtures of DPPC and DSPE and (e) SonoVue (control). Zeta potentials of the nanobubbles (f) and SonoVue (g).

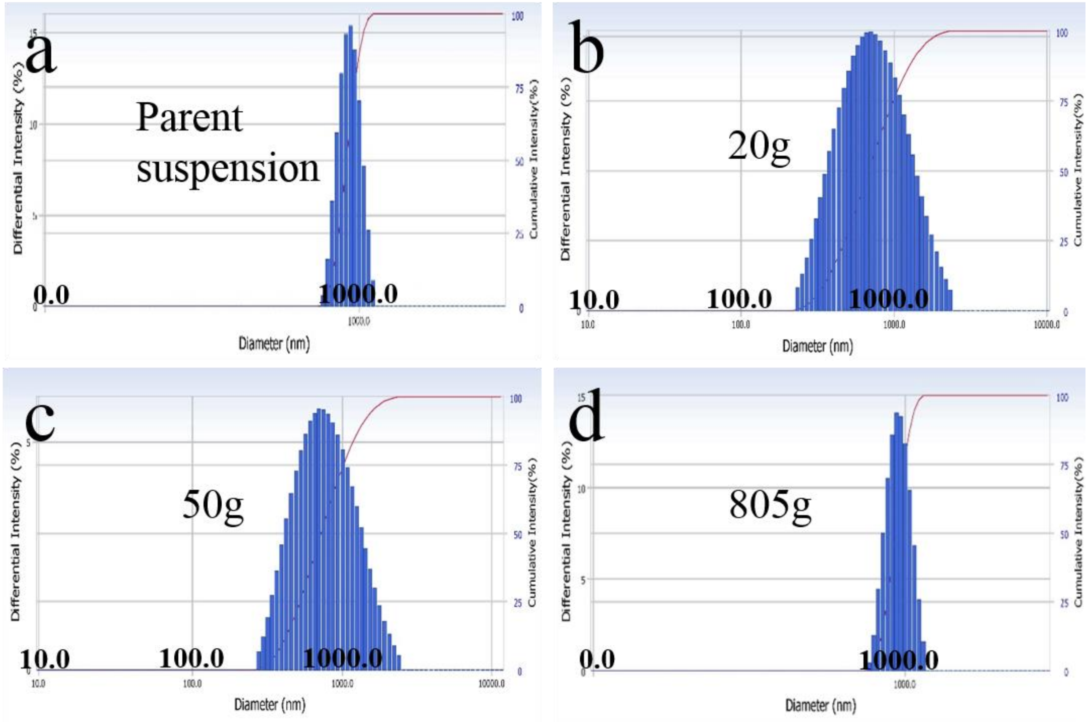

Supplementary Fig S2: Effect of centrifugation on the mixture of nanobubbles and microbubbles. (a) Diameter of the mixture of nanobubbles and microbubbles before centrifugation. Diameters of the bubbles prepared using centrifugation speeds of 20 g (b), 50 g (c) and 805 g (d).

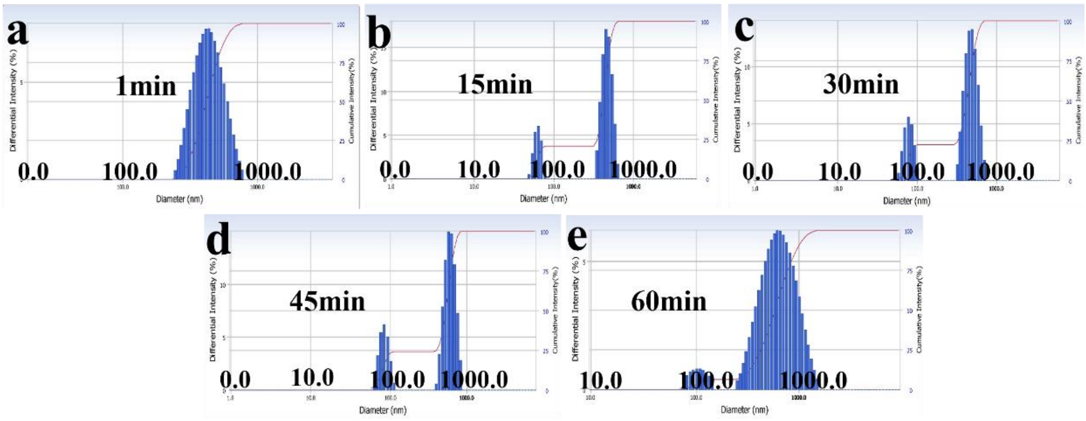

Supplementary Fig S3: Diameter distributions of nanobubbles after storage at 25 ℃ for 1 min (a), 15 min (b), 30 min (c), 45 min (d) and 60 min (e).
